# Genes Regulating Spermatogenesis and Sperm Function Associated With Rare Disorders

**DOI:** 10.3389/fcell.2021.634536

**Published:** 2021-02-16

**Authors:** Emma Linn, Lillian Ghanem, Hanisha Bhakta, Cory Greer, Matteo Avella

**Affiliations:** Department of Biological Science, College of Engineering and Natural Sciences, University of Tulsa, Tulsa, OK, United States

**Keywords:** sperm motility, acrosome, Sertoli, centrosome, infertility, genetic disease

## Abstract

Spermatogenesis is a cell differentiation process that ensures the production of fertilizing sperm, which ultimately fuse with an egg to form a zygote. Normal spermatogenesis relies on Sertoli cells, which preserve cell junctions while providing nutrients for mitosis and meiosis of male germ cells. Several genes regulate normal spermatogenesis, some of which are not exclusively expressed in the testis and control multiple physiological processes in an organism. Loss-of-function mutations in some of these genes result in spermatogenesis and sperm functionality defects, potentially leading to the insurgence of rare genetic disorders. To identify genetic intersections between spermatogenesis and rare diseases, we screened public archives of human genetic conditions available on the Genetic and Rare Diseases Information Center (GARD), the Online Mendelian Inheritance in Man (OMIM), and the Clinical Variant (ClinVar), and after an extensive literature search, we identified 22 distinct genes associated with 21 rare genetic conditions and defective spermatogenesis or sperm function. These protein-coding genes regulate Sertoli cell development and function during spermatogenesis, checkpoint signaling pathways at meiosis, cellular organization and shape definition during spermiogenesis, sperm motility, and capacitation at fertilization. A number of these genes regulate folliculogenesis and oogenesis as well. For each gene, we review the genotype–phenotype association together with associative or causative polymorphisms in humans, and provide a description of the shared molecular mechanisms that regulate gametogenesis and fertilization obtained in transgenic animal models.

## Introduction

In sexual reproduction, female and male gametes, the egg and the sperm fuse to generate a new and unique embryo (Bhakta et al., 2019). Fertilization requires proper gametogenesis to ensure healthy, euploid and genetically intact sperm and eggs. At gametogenesis, chiasmata hold homologous chromosomes in opposition on the meiotic spindle during recombination to ensure accurate segregation in the haploid gametes, and defective chromosomal segregation results in embryonic lethality or developmental defects (Lu et al., 2012; Nagaoka et al., 2012). On the male side, spermatogenesis generates structurally defined sperm in the testes; spermatogonia stem cells are unipotent stem cells that self-renew or differentiate into spermatocytes which traverse the Sertoli cell blood–testis barrier to enter the seminiferous tubules (Law et al., 2019). Here, spermatocytes complete two meiotic divisions to generate round spermatids, and through spermiogenesis, size, shape, and organelle composition of spermatids undergo significant changes, which lead to the formation of fully elongated sperm. At this stage, sperm are immotile and unable to fertilize an egg *in vivo*; to complete their maturation process, sperm must transit through the caput and cauda epididymis, where they acquire the ability to become motile (Da Ros et al., 2015). Finally, in the female reproductive tract, sperm undergo capacitation, which consists of a series of physiological changes that enable the sperm to fertilize an egg. Capacitated sperm can undergo acrosome exocytosis, a step in fertilization that consists of the exocytosis of a sub-Golgi-derived vesicle, the acrosome, that surrounds the nucleus in the apical region of the sperm head. Capacitated sperm are also enabled to bind to the zona pellucida and fertilize the egg (Puga Molina et al., 2018; Ritagliati et al., 2018).

Each step described above is regulated by a number of genes whose targeted deletion in transgenic animal models has been shown to cause defective gametogenesis, fertilization and fertility phenotypes. Here, we review 21 different rare diseases associated with fertility disorders due to mutations in genes controlling molecular pathways that ensure proper gametogenesis and fertilization (Table 1). To identify the genetic intersection between defective spermatogenesis and rare diseases, we screened 680 rare genetic conditions affecting humans in the Genetic and Rare Diseases Information Center (GARD), and the Online Mendelian Inheritance in Man (OMIM). We then performed extensive literature search, to narrow down our list to 82 monogenic and 20 polygenic conditions presenting a fertility phenotype associated with the rare disorder. Among the papers screened, we looked specifically for phenotypes pertaining to testes, male germ cell development and differentiation, Sertoli cell development, spermatogonia, spermatogenesis, meiosis checkpoints, centriole, centrosome, acrosome biogenesis and exocytosis, manchette formation, nuclear sperm compaction, sperm head and tail development, sperm motility, sperm capacitation, and hyperactivation. We have identified 21 conditions, caused by deleterious mutations in 22 genes regulating different aspects of sperm development, maturation, and function. Two of these conditions were identified using the Clinical Variant (ClinVar) database (Greer et al., 2021) (Table 1). For each gene and the related disorder, we provide a description of the fertility phenotype, the underlying defective molecular mechanism, and the phenotypic characterization of genome-edited animal models.

**TABLE 1:** List of genes regulating different aspects of sperm development and function.

Gene	Description	Fertility Phenotype	Rare Genetic Disease
*ABCD1*	ATP binding cassette subfamily D member 1 [Source:HGNC Symbol;Acc:HGNC:61]	Sertoli-cell-only syndrome and azoospermia	X-Linked Adrenoleukodystrophy, Adrenomyeloneuropathy Type
*ATM*	ATM serine/threonine kinase [Source:HGNC Symbol;Acc:HGNC:795]	Meiosis arrest at gametogenesis: development of spermatocytes arrests between the zygotene and pachytene stages; meiosis was disrupted before normal arrest at prophase I and absence of primary oocytes and follicles	Ataxia telangiectasia
*BUB1B*	BUB1 mitotic checkpoint serine/threonine kinase B [Source:HGNC Symbol;Acc:HGNC:1149]	Impaired proliferation of spermatogonia, abnormal chromosome segregation in spermatocytes, abnormal MII oocyte chromosomal configurations, and meiotic chromosome segregation defects	Premature chromatid separation with mosaic variegated aneuploidy syndrome
*CATSPER2*	Cation channel sperm associated 2 [Source:HGNC Symbol;Acc:HGNC:18810]	Failure to undergo hyperactivation, failure to fertilize eggs	Deafness infertility syndrome
*CFTR*	CF transmembrane conductance regulator [Source:HGNC Symbol;Acc:HGNC:1884]	Defective sperm motility, oligospermia, asthenospermia and teratospermia	Cystic fibrosis due to CTFR mutations
*CLPP*	Caseinolytic mitochondrial matrix peptidase proteolytic subunit [Source:HGNC Symbol;Acc:HGNC:2084]	Severe defects in granulosa cell differentiation and disruption of normal oogenesis and defective cellular biogenesis during early spermatogenesis	Perrault Syndrome type III
*DCAF17*	DDB1 and CUL4 associated factor 17 [Source:HGNC Symbol;Acc:HGNC:25784]	Defective manchette formation, leading to aberrant sperm morphology and lower motility	Woodhouse Sakati syndrome
*DNAH1*	Dynein axonemal heavy chain 1 [Source:HGNC Symbol;Acc:HGNC:2940]	Abnormal sperm flagella morphology and defective sperm motility	Primary ciliary dyskinesia type 37
*FGD1*	FYVE, RhoGEF and PH domain containing 1 [Source:HGNC Symbol;Acc:HGNC:3663]	Aberrant sperm morphology, absence of the acrosome	Aarskog Syndrome
*GJA1*	Gap junction protein alpha 1 [Source:HGNC Symbol;Acc:HGNC:4274]	Interrupted folliculogenesis after the primary follicle stage, Sertoli-cell-only syndrome	Oculodentodigital dysplasia
*GPX4*	Glutathione peroxidase 4 [Source:HGNC Symbol;Acc:HGNC:4556]	Decreased sperm progressive motility associated with impaired mitochondrial membrane potential and cellular abnormalities (e.g., hairpin-like flagella bend at the midpiece, swelling of mitochondria) and oligoasthenozoospermia.	Spondylometaphyseal dysplasia, sedaghatian type
*HJV*	Hemojuvelin BMP co-receptor [Source:HGNC Symbol;Acc:HGNC:4887]	Spermatogenesis defects and hypogonadotropic hypogonadism	Hemochromatosis 2A
*NPHP4*	Nephrocystin 4 [Source:HGNC Symbol;Acc:HGNC:19104]	Reduced sperm count and motility defects	Nephronophthisis
*PKD1*	Polycystin 1, transient receptor potential channel interacting [Source:HGNC Symbol;Acc:HGNC:9008]	Cystic testis, Sertoli-cell-only syndrome and azoospermia	Autosomal dominant polycystic kidney disease
*PKD2*	Polycystin 2, transient receptor potential cation channel [Source:HGNC Symbol;Acc:HGNC:9009]	Cystic testis, Sertoli-cell-only syndrome and azoospermia, defective sperm migration towards eggs *in vivo* (in fruit fly)	Autosomal dominant polycystic kidney disease
*P0C1A*	POC1 centriolar protein A [Source:HGNC Symbol;Acc:HGNC:24488]	Defective spindle formation, defective meiosis, aneuploidy.	Short Stature, Onychodysplasia, Facial Dysmorphism, and Hypotrichosis Syndrome
*P0C1B*	POC1 centriolar protein B [Source:HGNC Symbol;Acc:HGNC:30836]	Immotile sperm with aberrant axoneme architecture	Cone Rod Dystrophy type 20
*PRKAR1A*	Protein kinase cAMP-dependent type I regulatory subunit alpha [Source:HGNC Symbol;Acc:HGNC:9388]	Round spermatids developing large nuclear areas devoid of chromatin, resulting in aberrant sperm morphology with defects in sperm head and tail and inability of sperm to bind to the zona pellucida or fuse with eggs.	Carney complex
*SLC22A5*	Solute carrier family 22 member 5 [Source:HGNC Symbol;Acc:HGNC:10969]	Anatomically-deformed epididymis and obstructive azoospermia	Acetyl-carnitine deficiency
*SLC29A3*	Solute carrier family 29 member 3 [Source:HGNC Symbol;Acc:HGNC:23096]	Oligospermia and secondary male infertility due to Sertoli-cell-only syndrome	Histiocytosis-lymphadenopathy plus syndrome
*SLC39A4*	Solute carrier family 39 member 4 [Source:HGNC Symbol;Acc:HGNC:17129]	Sperm motility possibly due to defective zinc uptake	Acrodermatitis enteropathica
*SMN1*	Survival of motor neuron 1, telomeric [Source:HGNC Symbol;Acc:HGNC:11117]	Disrupted spermatogenesis due to aberrant splicing in prepubertal testis	Adult-onset spinal muscular atrophy

*Deleterious variants in each of these genes have also been associated with monogenic rare disorders. Columns from left to right-(A) Gene name. (B) Class of protein produced by gene. (C) Observed fertilization or gametogenesis phenotype associated with gene mutation. (D) Genetic disorder associated with gene mutation.*

## Mechanisms Regulating Sertoli Cell Development and Function at Spermatogenesis

**FIGURE 1:**
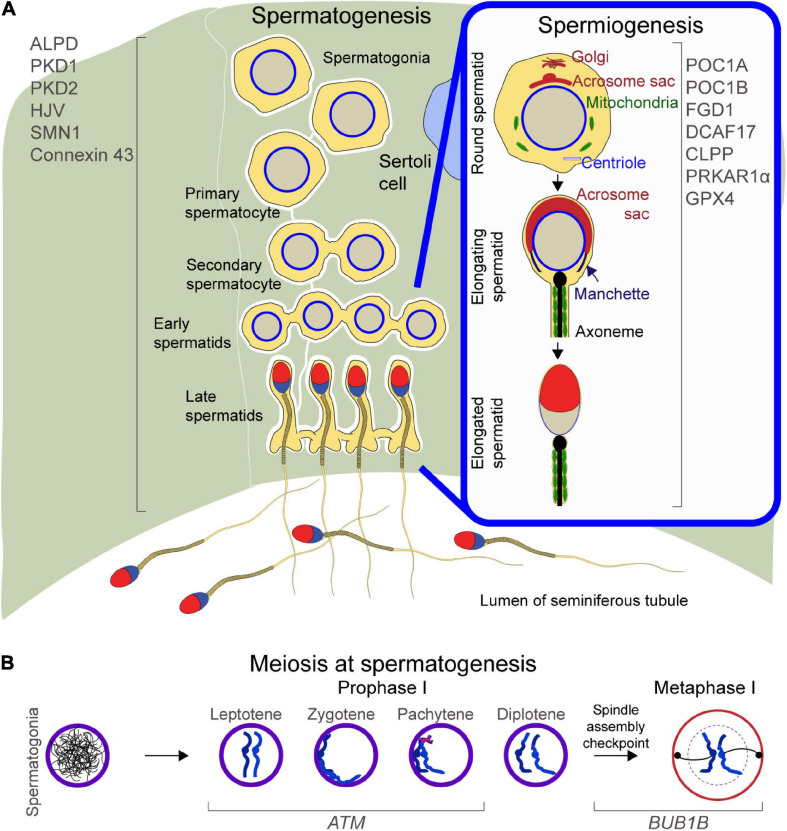
Schematic of spermatogenesis and spermiogenesis: **(A)** Left, proteins regulating spermatogenesis. Right inset, proteins mediating cellular remodeling at spermiogenesis. **(B)** Meiosis during spermatogenesis, with focus on Prophase and Metaphase II and proteins regulating chromosome migration, DNA double strand breaks repair, and spindle assembly checkpoint.

Spermatogenesis generates millions of haploid motile sperm from diploid spermatogonia and is a process mediated by a series of concerted molecular interactions between the developing germ cells and the somatic Sertoli cells (Law et al., 2019). Due to the presence of a significant amount of highly unsaturated fatty acids, the continuous cell proliferation, and high enzymatic activity, testes are subject to oxidative stress due to the overexpression of reactive oxygen species, which may compromise sperm count or affect sperm DNA integrity (Aitken, 2020). Key regulators of spermatogenesis are the peroxisomal membrane adrenoleukodystrophy protein (ALDP), the hemojuvelin gene *(HJV)*, Polycystic kidney disease (*PKD*) 1 and 2, the Survivor motor neuron 1 (*SMN1)*, which codes for the SMN protein, the Equilibrative Nucleoside Transporter 3 (ENT3), and Gap Junction Protein, Alpha-1 (GJA1), which codes for Connexin 43. ALDP regulates the degradation process of very long-chain fatty acids during spermatogenesis, HJV regulates iron metabolism in the testes, and PKD1 and 2 control male germ cell development by regulating the mTOR signaling pathway, while SMN, CONNEXIN 43 and ENT3 regulate normal Sertoli cell development, maturation and physiology (Figure 1A, left).

Very long chain fatty acids (VLCFAs) (fatty acids with C > 20) are constituents of cellular lipids (e.g., sphingolipids and glycerophospholipids) and serve as precursors of lipid mediators (van Roermund et al., 2008). VLCFAs converted to VLCFA-CoAs, are transferred into the cell’s peroxisomes, and are subjected to β-oxidization into long-chain or acyl-CoAs, which are transported to the mitochondria where they undergo β-oxidization (van Roermund et al., 2008). ALDP is encoded by the ATP Binding Cassette Subfamily D Member 1 (*ABCD1*) located on the X chromosome (Watkins et al., 1995). In humans, loss-of-function mutations in *ABCD1* affect the VLCFA degradation process, leading to a pathogenic accumulation of saturated C24–C26 VLCFAs in the plasma, brain, adrenal grand, and other tissues (Moser, 1997), causing myelopathies, as reported for the ***X-linked adrenoleukodystrophy (X-ALD)*** and the adult form, the Adrenomyeloneuropathy type (Moser, 1997). Typically, onset of adrenomyeloneuropathy type is around the age of 30 years, with patients presenting adrenocortical dysfunction, peripheral neuropathy, poor androgenization, low testosterone and elevated LH and FSH, lesions in interstitial cells of the testes, smaller seminiferous tubules, low ejaculate volumes, oligospermia, or azoospermia, which lead to male infertility (Powers and Schaumburg, 1981; Assies et al., 1997; Aversa et al., 1998). In testes, docosahexaenoic acid (C22:6n-3; DHA) synthesis in round spermatids from linolenic acid is controlled by ELOVL2 and ELOVL5 (Gregory et al., 2013, p. 2) and is mediated by peroxisomal β-oxidation (Rejraji et al., 2006). Mitochondrial and peroxisomal β-oxidation induce generation of reactive oxygen species, whose high levels have been associated with male infertility due to defective spermatogenesis and leukocytospermia (Pasqualotto et al., 2000). Of note, deletion of *Abcd1* in hemizygous male mice does not recapitulate the human phenotypes (Brennemann et al., 1997) and males are fertile (Lu et al., 1997), which raises the prediction of the presence of putative DNA variant modifier(s) in humans that would elicit the pathogenic phenotypes. In particular, an import machinery in peroxisomes regulates the transport of matrix proteins; PEX13 is a main component of this transport machinery and *Pex13* deletion leads to peroxisomal biogenesis defects in transgenic mice; more specifically, conditional knockout of *Pex13* in Sertoli cells leads to a “Sertoli-cell-only” syndrome with a significant increase in triglycerides and cholesteryl esters (Nenicu et al., 2009). Also, conditional deletion of *Pex13* in pre-meiotic germ cells impairs the import of peroxisomal matrix proteins in germ cells, leading to interruption of differentiation at the round spermatid stage and azoospermia in male mice (Brauns et al., 2019).

Hemojuvelin gene is involved in iron metabolism and encodes the hemojuvelin protein (Huang et al., 2005). In *Hjv* knockout mice, absence of the hemojuvelin protein causes a reduction in production of hepcidin mRNA. Hepcidin is a regulator of iron metabolism and is able to bind to ferroportin. Upon targeted disruption of *Hjv*, increased levels of ferroportin expression have been reported in several tissues including the liver, spleen, and blastolateral membrane (Huang et al., 2005). Ferroportin is an ion transporter present in cellular membranes of macrophages and endothelial cells of the intestines, and it plays a significant role in nutrient absorption. Iron is transported through cellular membranes by ferroportin, and iron uptake is downregulated by hepcidin binding, indicating that *Hjv* plays a role in preventing iron overload by maintaining physiological hepcidin levels (Huang et al., 2005; Niederkofler et al., 2005). In humans, missense mutations in *HJV* are associated with ***Juvenile Hemochromatosis***, which manifests in teens and young adults with high iron levels throughout the entire body and consequent symptoms such as cardiac deficits, diabetes, and hypogonadism (Papanikolaou et al., 2004). Excess iron levels are observed in knockout *Hjv* mice, particularly in the kidney, liver, heart, pancreas, and testis (Huang et al., 2005). However, cardiac and organ dysfunction, diabetes, hepatic fibrosis, and fertility phenotypes are not observed as are in human hemochromatosis (Huang et al., 2005). Male patients with hemochromatosis experience fertility disorders due to spermatogenesis defects and hypogonadotropic hypogonadism (McDermott and Walsh, 2005; Leichtmann-Bardoogo et al., 2012). Excess iron levels cause oxidative stress, a phenomenon that can hamper the delicate homeostasis required for spermatogenesis (Leichtmann-Bardoogo et al., 2012). Spermatogenesis is protected from iron imbalances by Sertoli cells (Leichtmann-Bardoogo et al., 2012). Sertoli cells store excess iron and redeposit it into new spermatocytes (Leichtmann-Bardoogo et al., 2012). Mice with high iron levels generated by deletion of the genes encoding iron regulatory protein 2 (IRP2) and Human homeostatic iron regulator protein (HFE) demonstrate iron buildup around the seminiferous tubules, but lower buildup within the tubules, observations that indicate a more autonomous iron regulatory system during spermatogenesis (Leichtmann-Bardoogo et al., 2012). Homozygous knockout *Irp2* mice contain viable sperm in the epididymis and demonstrate normal fertility (Leichtmann-Bardoogo et al., 2012). The same amount of apoptotic cells are observed in knockouts as are wild type mice (Leichtmann-Bardoogo et al., 2012). Observations of sperm were not made and no subsequent studies have utilized this model for iron regulation in the testis. Additionally, the most frequent non-diabetic endocrine symptom of hemochromatosis presents as hypogonadotropic hypogonadism (McDermott and Walsh, 2005). Hypogonadism is caused by accumulation of iron in the pituitary gonadotroph cells, which are responsible for regulating gonadotropins (McDermott and Walsh, 2005). Low levels of TSH, LH, and testosterone manifest, resulting in variable fertility defects (McDermott and Walsh, 2005).

*PKD 1* and *2* are two protein-coding genes associated with the insurgence of ***Autosomal dominant polycystic kidney disease (ADPKD)***, a monogenic disorder that results in the bilateral development of renal cysts, leading to end-stage renal disease (Torres et al., 2007). *PKD1* codes for Polycystin-1, an integral membrane protein presenting 11 transmembrane domains and an extracellular region that includes 12 PKD immunoglobulin-like fold domains, known as PKD domains, which typically function as mediators for protein-protein or protein–carbohydrate interactions (Hughes et al., 1995; Mochizuki et al., 1996). *PKD2* codes for Polycystin-2, a non-selective cation/calcium channel. Polycystins form a complex that regulates intracellular levels of Ca^2+^ in different cell types involved in cell–cell and cell–matrix interactions, in the endoplasmic reticulum, or in primary cilia. In primary cilia, the intraflagellar transport motor component KIF3A mediates protein movement in the cilium and is necessary for ciliary formation (Harris and Torres, 2009). Kidney-specific deletion of *Kif3a* disrupts this protein transport and mutant mice develop renal cysts (Lin et al., 2003). Moreover, the polycystin complex serves as a flow-sensor in the cilium mediated by Ca^2+^ influx into the cell that occurs through the polycystin-2 channel. *Pkd1* and *Pkd2* null mice are embryo lethal, due to defects in formation of the kidney, pancreas, heart, and capillary blood vessels, whereas conditional deletion of *Pkd1* leads to the development of cysts in the mouse kidneys (Shibazaki et al., 2008). *Pkd2*^ws25/–^ mice (an animal model of ADPKD) show hepatic cysts, cardiac defects, and renal failure (Wu et al., 2000). Extra-renal cyst development occurs in male reproductive organs including the testis, epididymis, seminal vesicles, and prostate (Danaci et al., 1998).

In adult human testes, presence of cilia has been reported in the peritubular myoid cells and in differentiating Leydig cells. Of note, cilia are also present in pathological conditions such as in azoospermic patients with Klinefelter syndrome (presenting Sertoli cell-only phenotype) (Nygaard et al., 2015). Atrophic testes typically present peritubular cells that produce aberrant-long cilia (Nygaard et al., 2015). Male fertility phenotypes are not uncommon in ADPKD patients, with individuals presenting necrospermia and testis cysts; one possible molecular mechanism behind such fertility phenotypes may reside within the mechanistic Target of Rapamycin (mTOR) signaling pathway. The mTOR signaling is aberrantly upregulated in ADPKD and rapamycin has been shown to inhibit cyst expansion (Shillingford et al., 2006). In testes, mTOR regulates spermatogonial stem cell maintenance and differentiation, controls the physiology of Sertoli cells, and helps preserve the maintenance of the blood–testis barrier, while nurturing maturing sperm during spermatogenesis (Moreira et al., 2019). Indeed, chronic inhibition of mTOR by rapamycin leads to a partially reversible spermatogenic arrest in adult male mice; this is due to defects in sex body formation and meiotic sex chromosome inactivation, which lead to partially reversible impaired spermatogenesis and male infertility (Zhu et al., 2019). Dysregulation of mTOR signaling in the testes of ADPKD patients could represent the cause of cystic testes and possibly other unreported testis-related fertility phenotypes.

*SMN1* codes for the SMN protein, which mediates the assembly of small nuclear ribonucleoproteins (snRNPs), key constituents of the spliceosome machinery (Lefebvre et al., 1995). SMN is expressed in motor neurons, as well as muscle, heart, lung, and intestine tissues (Ottesen et al., 2016). In humans, deletion and missense mutations have been associated with the insurgence of ***Adult Spinal Muscular Atrophy (SMA)***, a group of genetic conditions that gradually abolishes motor neurons that result in muscle weakness, impairment in the control of skeletal muscle activities (e.g., speaking, walking, breathing, and swallowing), and atrophy (Lefebvre et al., 1995). Typically, the severity of SMA is defined by the SMN levels: SMN signals the proper assignment of spliceosomal proteins to the corresponding snRNA (Pellizzoni et al., 2002). Subsequently, SMN dissociates from this complex and the snRNA proceeds to RNA splicing (Pellizzoni et al., 2002). In the mammalian genome, the paralog *SMN2* is unable to rescue the phenotypes due to *SMN1* null mutations (because of skipping of exon 7 at splicing that results in an only partially functioning SMNΔ7 truncated protein) (Lorson et al., 1999). Abnormal splicing of particular genes such as Neurexin 2 *(NRXN2)*, which coordinates synapse development, and Ubiquitin like modifier activating enzyme 1 *(UBA1)*, whose function is to regulate ubiquitin levels, have been proposed to trigger neuromuscular specific phenotypes observed in SMA (See et al., 2014; Wishart et al., 2014).

Homozygous *Smn1 Smn2* double knockout mice present periimplantation lethality (Hsieh-Li et al., 2000). Therefore, extensive research efforts have been made to establish a mouse model with an intermediate form of the disease, in an effort to provide a research tool to better investigate SMA. To this end, expression of human *SMN2* and *SMNΔ7* in the *Smn1* null background in transgenic mice is shown to rescue the embryo-lethal phenotype, with transgenic rescue mice showing spinal cord and skeletal muscle abnormalities similar to phenotypes seen in SMA patients (Hsieh-Li et al., 2000). Another SMA mouse model known as *Smn*^C/C^ mouse, expresses two copies of a chimeric-hybrid transgene defined by the murine genomic *Smn1* (exons 1-6) and human genomic *SMN2* (spanning exons 7–8) carrying a 42 kb genomic *SMN2* segment (downstream of exon 8) (Osborne et al., 2012). This mouse generates ∼25–50% of the SMN protein, and presents symptoms of SMA, including small testis, reduced number of post-meiotic cells, and disrupted spermatogenesis (Ottesen et al., 2016). Also, sperm count has been reported as severely reduced (∼10 times lower than normal mice) (Ottesen et al., 2016). *Smn*^C/C^ testis transcriptome analyses report downregulation of genes expressed in late spermatocytes and spermatids, and an altered expression of genes regulating apoptotic pathways, which may affect pre-pubertal Sertoli cell development (Bao et al., 2015).

The Solute Carrier Family 29 Member 3 (*SLC29A3*) gene encodes ENT3, an intracellular nucleoside transporter. ENT3 localizes to late endosomes, lysosomes, and mitochondria, and it translocates hydrophilic nucleosides across the membrane to regulate DNA synthesis and purinergic signaling (Young et al., 2013, p. 29). In humans, homozygous or compound heterozygous loss of function mutations in *SLC29A3* have been associated with ***Histiocytosis-lymphadenopathy plus syndrome***, a genetic disease that includes 4 previously thought different histiocytic conditions. Resulting conditions include Faisalabad histiocytosis (FHC), Sinus Histiocytosis with Massive Lymphadenopathy (SHML), H syndrome, and Pigmented Hypertrichosis with Insulin-dependent Diabetes mellitus syndrome (PHID) with a broad spectrum of clinical defects involving the skin, pancreas, eyes, musculoskeletal system, or presenting multiple hematological and endocrinological features (Morgan et al., 2010). In particular, H syndrome has been reported to associate with hyperpigmentation, hypertrichosis, hepatosplenomegaly, heart anomalies, hearing loss, low-height, hyperglycemia and hypogonadism (Molho-Pessach et al., 2008, p. 3). Gene deletion of *Slc29a3* in mice recapitulates the defective human phenotypes including hypogonadism (Nair et al., 2019); male mice show severe subfertility, producing small litters (2/3 pups per litter) and secondary infertility, possibly due to endocrinopathy (Nair et al., 2019), although no phenotypic evidence for the mouse gametes is reported. Of note, ENT3 is expressed in mammalian Sertoli cell lines (Kato et al., 2006), implicating a possible role of ENT3 in mammalian spermatogenesis.

*GJA1* codes for Connexin 43, a main component of gap junctions, which consists of intercellular channels and ensures diffusion of low-weight molecules between adjacent cells. *GJA1* is expressed in a broad variety of tissues such as brain, eye, muscles, skin, and bones. More than 70 different missense *GJA1* mutations have been associated in humans with misassembled channels or altered channel conduction properties, leading to a genetic condition known as ***Oculodentodigital Dysplasia***, an autosomal dominant disorder with high penetrance and variable expressivity (Paznekas et al., 2003). Typical phenotypes include craniofacial anomalies (e.g., thin nose with hypoplastic alae nasi, small anteverted nares, prominent columnella, and microcephaly), brittle nails and hair abnormalities of hypotrichosis, dysplastic ears and conductive hearing loss, ophthalmic defects (e.g., glaucoma, and optic atrophy), cleft palate, and mandibular overgrowth (Paznekas et al., 2003). Gene deletion in transgenic mice leads to perinatal lethality due to congenital defects in the heart (Reaume et al., 1995, p. 43). Conditional deletion of *Gja1* in mouse testis results in seminiferous tubules presenting only Sertoli cells which surprisingly continue to proliferate even after 20 days of age, and severe reduction of maturing germ cells lead to a Sertoli-cell-only phenotype (Sridharan et al., 2007).

## Checkpoint Signaling Pathways Regulate DNA Damage Response at Meiosis

To ensure successful transmission of genetic information to the progeny, meiosis checkpoints halt the beginning of late cell-cycle events until the completion of earlier events (Hartwell and Weinert, 1989). Checkpoints are necessary to activate repair mechanisms upon DNA damage, for genome stability, and to ensure faithful segregation of replicated chromosomes (Hartwell and Weinert, 1989). At meiosis, the Ataxia-Telangiectasia Mutated *(ATM)* and the Bub1-Mitotic Checkpoint Serine/Threonine Kinase B (*BUB1B)* genes regulate cell cycle checkpoint signaling pathways (Figure 1B).

*ATM* encodes a kinase that responds to DNA double strand breaks (DSBs) by regulating the ATM- and Rad3-related (ATR) kinase, a checkpoint kinase required for meiosis progression (Jazayeri et al., 2006; Lange et al., 2011) and for DNA repair in cases of excess DSBs formation (Lange et al., 2011). In humans, missense, frameshift, or nonsense mutations in the *ATM* gene result in the absence of a functional ATM protein and the insurgence of a rare genetic disease defined as***Ataxia telangiectasia*** (Jacquemin et al., 2012). Symptoms include susceptibility to cancer development, cerebellar impairments causing ataxia, oculomotor apraxia, immune deficits, and fertility impairments (Jacquemin et al., 2012). Gonadal insufficiencies causing infertility originate with meiotic arrest, and meiotic disruptions likely occur in the crossing over stages of prophase I (Xu et al., 1996).

*Atm* null mice lack round and elongated spermatids (Xu et al., 1996), whereas in females degenerate ovaries do not contain early stage oocytes or development of follicles (Xu et al., 1996). *Atm* is epistatic over the Spo11 Initiator Of Meiotic Double Stranded Breaks gene (*Spo11*), which regulates the formation of DSBs at crossing over (Lange et al., 2011). *Atm* null spermatocytes present an increase in SPO11 expression, likely associated with a higher number of DSBs (Lange et al., 2011). It is believed that the ATM kinase is activated by DSBs, and by phosphorylation it downregulates SPO11, decreasing the generation of DSBs in a negative feedback loop (Lange et al., 2011). *Atm* knockout mice show growth retardation, immune deficits, develop thymic lymphomas, and present testicular and ovarian abnormalities (Xu et al., 1996). Immunostaining of spermatocytes with polyclonal antibodies against the chromosomal core protein COR1 has been performed to follow chromosomal synapse complexes during crossing over (Xu et al., 1996). At meiosis, *ATM* initiates repair of DSBs (Xu et al., 1996; Lange et al., 2011) and absence of ATM is found to correlate with unusually high numbers of univalents, only partially synapsed bivalents, and a delay in observed synapsis (Xu et al., 1996). Also, bivalents are fragmented, causing chromosome fragmentation. In the testes of *Atm* null males, development of spermatocytes halts between the zygotene and pachytene stages of meiotic prophase, which lead to male infertility (Xu et al., 1996). Null mice present a female fertility phenotype as well: in the null ovaries, no primary oocytes or follicles are observed and meiosis is disrupted before normal arrest at prophase I, which results in female infertility (Xu et al., 1996).

*BUB1B* codes for a kinase controlling spindle checkpoint function: BUB1B delays the onset of anaphase by acting at the kinetochore and inhibiting the anaphase-promoting complex/cyclosome; this mechanism ensures proper chromosome segregation prior to cell division (Cahill et al., 1998; Taylor et al., 1998; Perera et al., 2007). In humans, deleterious biallelic mutations (missense or frameshift) which inactivate the BUB1B kinase domain are associated with a ***Premature Chromatid Separation Trait***, which leads to***Mosaic Variegated Aneuploidy Syndrome*** (Limwongse et al., 1999). This autosomal recessive disorder is defined by mosaic aneuploidies (trisomies and monosomies) observed in different chromosomes and tissues. Patients with this condition present aneuploidies in more than 25% of their cells (Rudd et al., 1983), which is associated with variable developmental delay and a wide range of other congenital defects such as intrauterine growth retardation, microcephaly and eye anomalies. Plus, severe risks of malignancy are reported including rhabdomyosarcoma, Wilms tumor, leukemia (Hanks et al., 2004) and ovarian cancer (Feng et al., 2019). Furthermore, *BUB1B* has been associated with infertility due to premature ovarian insufficiency (Chen et al., 2020). Gene deletion of mouse *Bub1b* leads to embryo lethality (Baker et al., 2004); however, through a sophisticated gene deletion strategy, mutant mice homozygous for the ‘H’ allele (*Bub1b*^H/H^) were generated and found to be infertile. In these mice, transcription of the ‘H’ allele leads to a precursor mRNA which is occasionally spliced abnormally (Baker et al., 2004), causing the development of mutant mature *Bub1b* mRNA that is not translated; this reduces the cellular BUB1B protein levels (Baker et al., 2004), leading to male and female infertility. Testis weight of homozygous mutant males is lower than normal controls and sperm count is significantly reduced. Sperm motility, morphology, and ability to bind to the zona are reported as normal, though no data in the original manuscript is shown regarding sperm ability to fuse with the ovulated eggs. Of note, *Bub1b*^H/H^ ovulates a larger number of eggs (206 eggs) compared to normal (152), but only 6% lead to 2-cell stage embryos (Baker et al., 2004). Also, 5% of spermatocytes in metaphase of meiosis II present abnormal karyotypes; moreover, in follow-up studies, conditional deletion of *Bub1b* in mouse testis impairs proliferation of spermatogonia, induces abnormal chromosome segregation in spermatocytes, and drastically reduces sperm production (over 80%), resulting in male infertility (Perera et al., 2007). Hence, lower expression or full deletion of *Bub1b* in the testes impairs spermatogenesis and fertilization (Baker et al., 2004). In females, *Bub1b*^H/H^ ovaries are able to ovulate MII oocytes, though ∼70% of ovulated eggs present highly abnormal MII chromosomal configurations, leading to meiotic chromosome segregation defects and female infertility (Baker et al., 2004).

## Sperm Cellular Organization and Shape

In the final stage of spermatogenesis, defined as spermiogenesis, spermatids undergo cellular and nuclear reshaping, organelle reorganization, and tail formation, which results in the development of mature spermatozoa. Centrosomes are formed from centrioles, organelles located near the nuclear envelope of animal cells which are necessary for the proper organization of microtubules, as well as for the positions of the nucleus and other organelles (Avidor-Reiss and Turner, 2019). At spermiogenesis, the manchette, a transient microtubular platform consisting of α- and β-tubulin heterodimers (Lehti and Sironen, 2016), mediates a number of considerable changes in the developing haploid germ cells, which include the development of the sperm tail as well as the condensation and elongation of the sperm head (Gaffney et al., 2011). Mature mammalian sperm are characterized by a strong flagellum to drive them through the female genital tract and are equipped with several mitochondria that generate energy for the flagellum motility (Gaffney et al., 2011); the flagellum axoneme is defined by two central singlet microtubules surrounded by nine microtubule doublets, which in turn are enclosed by nine outer dense fibers (Gaffney et al., 2011). The sperm head presents a condensed haploid nucleus containing tightly packed DNA coiled around protamines (Wykes and Krawetz, 2003). Underlying the anterior plasma membrane of the sperm head is the acrosome, a secretory Golgi-derived subcellular organelle, that contains a number of lytic enzymes, which are not necessary for binding or penetration of the zona pellucida in mice (Buffone et al., 2014). Nonetheless, one of these enzymes, Acrosin, is necessary for zona penetration in hamsters (Hirose et al., 2020) (Figure 1A, right panel).

Key molecular players in the definition of cellular structure are POC1 Centriolar Proteins (POC1) A and B (*POC1A* and *B*), FYVE, RhoGEF and PH domain containing 1 (*FGD1* encoding FGD1), DDB1– and Cul4–Associated Factor 17 (*DCAF17*, encoding Cullin-RING E3), Caseinolytic Peptidase P (*CLPP*), and Protein Kinase Camp-Dependent Type I Regulatory Subunit A (*PRKAR1α*). The POC1 Centriolar Protein *(POC1)* A and B (*POC1A* and *B)* play important roles in the elongation and structural stability of the centriole (Geister et al., 2015). FGD1 possibly controls acrosome biogenesis, Cullin-RING E3 regulates manchette formation and nuclear compaction, CLPP regulates mitochondrial activity during early spermatogenesis, and *PRKAR1α* controls normal sperm head and tail development during male germ cell differentiation.

In human sperm, centrosomes are defined by the proximal centriole (PC), surrounded by the pericentriolar material (PCM), and the distal centriole (DC). The sperm DC is located at the base of the axoneme, and an atypical structure is defined when its microtubules are wide-opening outward. During spermatid development, a number of DC centriolar proteins organize into rods (Fishman et al., 2018). In bovine sperm, it has been shown that DC recruits the PCM, to establish a daughter centriole, which localizes to the spindle pole, while remaining attached to the axoneme (Fishman et al., 2018). This structure is an atypical sperm centriole, which serves as the second centriole of the zygote (Fishman et al., 2018). In humans, missense mutations or deletion of *POC1A* are associated with ***Short Stature, Onychodysplasia, Facial Dysmorphism, and Hypotrichosis Syndrome***, which results in stunted bone and ectodermal tissue development (Sarig et al., 2012). Deleterious mutations in *POC1A* result in an increased number of centrioles and an aberrant Golgi stack assembly, leading to abnormal cellular material trafficking (Sarig et al., 2012). Abnormal Golgi function has been associated with disorders of the bone and skin, and abnormal centriole formation causes severe disruption to Golgi trafficking (Sarig et al., 2012). Ciliary function also regulates chondrocytes differentiation, and *POC1A* deleterious mutations alter this process (Geister et al., 2015). In mice, expression of *Poc1a* is enriched in the seminiferous tubules and Sertoli cells, and LINE-retrotransposon mediated deletion of *Poc1a* (chagun mice) mimic the human disorder and results in male infertility, due to defective meiosis in germ cells (Sarig et al., 2012; Geister et al., 2015). Mutant mouse embryos develop additional centrosomes, and occasionally 3– 4 spindle poles, which likely results in aneuploidy (Geister et al., 2015). In cases where proper spindle formation proceeds, only ∼28% of cells develop primary cilia (Geister et al., 2015). While some spermatocytes are observed in knockout seminiferous tubules, developing germ cells are not generally present (Geister et al., 2015, p. 1). Particularly, in mutant males, secondary spermatocytes and round/elongating spermatids do not develop, which may indicate that cell death occurs in the pre-leptotene stage of spermatogenesis (Geister et al., 2015). Male infertility is due to these early meiotic defects originating with spindle formation (Geister et al., 2015, p. 1).

*POC1B* together with *POC1A*, maintains centriole structure and ensures proper formation of the mitotic spindle. Studies performed in zebrafish localize *poc1b* expression to the basal bodies of primary cilia in the retina membrane (Roosing et al., 2014). *POC1B* interacts with the FAM161 centrosomal protein A gene *(FAM161A)* to regulate ciliary development in the basal body (Roosing et al., 2014). Knockdown of *poc1b* is induced through a morpholino-oligonucleotide in zebrafish, which prevents the splicing or translation of Exon 2 (Roosing et al., 2014). Truncated poc1b protein interacts significantly less with fam161a and small eyes develop (Roosing et al., 2014). Additional phenotypes commonly observed with ciliopathies are displayed, including a curved body axis and kidney cysts (Roosing et al., 2014). In the free-living ciliate *Tetrahymena thermophila*, and in humans, POC1B is localized to the basal body cartwheel, to the site of new basal body assembly, and to the microtubule cylinder walls; this protein preserves the stability of basal bodies, while ensuring proper ciliary-based motility, and cilia formation (Pearson et al., 2009). *Poc1b* small interfering (si)RNA treated cells demonstrate a 30% decrease in ciliogenesis, alongside shortened and bent cilia development (Pearson et al., 2009). In humans, deletion or missense mutations in *POC1B*, usually affecting exons 6 or 7, cause the loss of association of the basal body to the primary cilium (Roosing et al., 2014). These mutations are associated with autosomal ***Cone Rod Dystrophy type 20*** (Roosing et al., 2014). Defects originating in the ciliary function of retina cause either complete absence of, or the progressive degeneration of, cone function affecting color detection (Roosing et al., 2014). Disease progression may later impair rod function, likely owing to degeneration of cone function (Roosing et al., 2014).

In *Drosophila*, *poc1b* is expressed in spermatogonia and throughout spermatogenesis (Khire et al., 2016). Unlike other ciliary regulatory proteins, POC1B has been found to be upregulated in the maturation of spermatogonia to spermatozoa (Khire et al., 2016). The giant centriole (GC) and the proximal centriolar-like structure (PCL) are two centrioles highly involved in proper centrosome formation (Khire et al., 2016). Downregulation of GC and PCL proteins is observed as spermatids mature to spermatozoa, whereas poc1b is consistently upregulated (Khire et al., 2016). Additionally, POC1B is highly expressed at the center of the PCL (Khire et al., 2016). In spermatocyte, POC1A and B are necessary for GC elongation; in particular, POC1A is shown to be necessary and sufficient for sperm motility, and POC1B for controlling the protein composition PCL (Khire et al., 2016). *Poc1b* mutants show immotile sperm presenting with aberrant axoneme architecture, and early embryos present monopolar spindles lacking microtubule aster (Khire et al., 2016). These mutant phenotypes are rescued by the expression of a *Poc1* transgene. In particular, the transgene consists of a *BamGal4* promoter which can drive the expression of a polycistronic mRNA consisting of an untagged *poc1A* and a GFP-tagged *poc1b* (Khire et al., 2016), specifically in late spermatogonia and early spermatocytes. Trangenic-rescue sperm can fertilize normal oocytes, and embryos show bipolar spindles and microtubule asters (Khire et al., 2016).

The *FGD1* gene encodes the FGD1 protein, a guanine-nucleotide exchange factor (GEF) that is responsible for the activation of the p21 GTPase CDC42 (German Pasteris et al., 1994; Zheng et al., 1996; Kierszenbaum et al., 2003). The FGD1 protein is defined by a proline-rich N-terminus, the GEF and pleckstrin homology (PH) domains, a FYVE-finger domain, and a PH domain (PH2) located at the C terminus of the protein; these latter domains mediate protein signaling and subcellular localization (Zheng et al., 1996). In humans, several mutations in *FGD1* have been associated with the manifestation of ***Aarskog-Scott syndrome*** (Lebel et al., 2002; Orrico et al., 2005; Bottani et al., 2007), an X-linked genetic disease defined by severe a short stature, hypertelorism, shawl scrotum, and brachydactyly, and occasionally intellectual disability (German Pasteris et al., 1994). Moreover, a 31 years old male patient with Aarskog-Scott syndrome is reported with infertility and recurrent miscarriage, possibly due to severe spermatogenesis defects, with 95% of his sperm lacking the acrosome (Meschede et al., 1996). In mice, *Fgd1* expression is localized at sites of active bone formation within osteoblasts, playing a pivotal role during skeletal formation (German Pasteris et al., 1994). In particular, *Fgd1* expression has been described in the subcortical actin cytoskeleton and in the Golgi apparatus (Estrada et al., 2001), the latter defining spermatid polarity and being the source of proacrosomal vesicles during the biogenesis of the acrosome (Kierszenbaum et al., 2003). Although no sperm-specific conditional knockout mouse for *Fgd1* has been reported yet, it is conceivable to speculate a role of *Fgd1* during acrosomal biogenesis (Meschede et al., 1996). Indeed, a conditional knockout mouse for *Fgd1* would prove useful to inform on the relation between CDC42 and FGD1 at spermatogenesis.

*DCAF17*, a member of DCAF family genes, encodes substrate receptor proteins for Cullin-RING E3 ubiquitin ligases, enzymes that act as key mediators of post-translational protein regulation in multiple cellular processes. Deleterious variants in *DCAF17* have been identified as the primary cause of ***Woodhouse Sakati syndrome***, a rare autosomal recessive genetic condition characterized by alopecia, hearing impairment, diabetes mellitus, learning disabilities, extrapyramidal manifestations, and hypogonadism (Alazami et al., 2008). Gene deletion of *Dcaf17* in male mice leads to infertility due to aberrant sperm development and morphology due to a defective manchette formation and nuclear compaction during spermiogenesis (Ali et al., 2018, p. 17): *Dcaf17* null males produce a low number of sperm that present abnormal shape and lower motility. Morphology analyses show sperm with deformed and decondensed nuclei with defective nuclear chromatin condensation, with triangular, oval or amorphous head shapes, miss-localized and deformed and detached acrosomes (which were missing in 20% of the sperm), disorganized and disrupted mitochondrial sheaths with disorganized outer dense fibers, and aggregating mitochondria near the deformed nucleus (Ali et al., 2018, p. 17), a phenotype similar to oligoasthenoteratozoospermia in humans. DCAF17 acts as receptor for several Cullin4-based E3 ligase complexes and serves as substrate to the E3 ligase complex for protein ubiquitination (Lee and Zhou, 2007). During sperm development, two proteins, CUL4A and CUL4B function as scaffold proteins for the assembly of the Cullin4-based E3 ligase complex (Jackson and Xiong, 2009). Testicular phenotypes obtained upon gene deletion of *Cul4a* and *Cul4b* resemble the results observed in Dcaf17 knockout testes; this may implicate a collaborative role of CUL4A, CUL4B, and DCAF17 proteins in regulating spermatogenesis via ubiquitin-mediated mechanism for maintenance of protein homeostasis.

*CLPP* encodes a mitochondrial peptidase conserved in prokaryotes and eukaryotes (Gispert et al., 2013) that is sufficient to digest small polypeptides without ATP requirement (Gispert et al., 2013). For hydrolysis of larger proteins, CLPP rings are secured by the activity of the ATP-dependent Clp protease ATP-binding subunit Clpx (CLPX) enzyme (Voos, 2009; Gispert et al., 2013). CLPP has been identified also as a component of mitochondrial unfolded protein response, a reaction in which CLPP expression increases under accumulation of unfolded proteins (Voos, 2009). The CLPP/CLPX protein complex establishes a proteolytic barrel structure in which target-proteins in the mitochondria are broken down (Gispert et al., 2013). In the mouse, *Clpp* expression is enriched in a variety of tissues, including liver, brain, heart, ovary, and testis (Gispert et al., 2013). In humans, missense *CLPP* mutations cause ***Perrault syndrome type 3***, which is characterized by sudden sensorineural hearing loss, epilepsy, growth retardation (Dursun et al., 2016), ovarian dysgenesis manifesting in amenorrhea, and azoospermia in men (Gispert et al., 2013; Demain et al., 2017). The missense mutations reported likely affect the alpha helices and beta sheets of CLPP, as well as disrupt the hydrophobic cleft of the protein that is responsible for the interaction with CLPX (Jenkinson et al., 2013). Consequent structural abnormalities cause deficits in CLPX functionality, resulting in decreased degradation of misfolded proteins. This excess of abnormal protein content induces poor cell functionality in expressed locations, including sensory hair follicles and developing ovaries (Jenkinson et al., 2013). *Clpp* null mice showed an increased expression of CLPX in the liver, brain, heart, testis, and ovary (Gispert et al., 2013). Analogous to the observed symptoms associated with *CLPP* mutations, increased CLPX expression has been correlated to impaired hearing, motor deficits, and male and female infertility (Gispert et al., 2013). *Clpp* deletion in mouse testes have been shown to lead to halted spermatogenesis before the formation of spermatids, likely due to the presence of misfolded proteins (Gispert et al., 2013; Jenkinson et al., 2013). It is conceivable that absence of CLPP leads to a compensatory effect by inducing the expression of cytosolic protease subunits to enhance degradation. Yet, *Clpp* null seminiferous tubules completely lack spermatids and mature spermatozoa in the testis, and electron microscope analysis shows severe defective cellular organization with no acrosomes or axonemes (Gispert et al., 2013).

*PRKAR1A* encodes the R1α regulatory subunit of cAMP-dependent protein kinase A (PKA). PKA mediates cellular cAMP signaling in mammals. PKA consists of a tetramer defined by two catalytic subunits and two regulatory subunits, which reversibly bind to cAMP to release the active catalytic subunits (Bossis and Stratakis, 2004). cAMP/PKA mediated-phosphorylation regulates cellular metabolism, proliferation, differentiation, and apoptosis (Bossis and Stratakis, 2004). *PRKAR1A* acts as a tumor suppressor gene, and frameshift mutations result in haploinsufficiency of R1α associated with ***Carney complex*** (Casey et al., 2000). This rare genetic disease is defined by different benign neoplasia, which affects a broad spectrum of tissues including heart, skin, and endocrine tissues, or results in abnormalities of skin complexion and a spotty appearance (Bossis and Stratakis, 2004). Mutant mice carriers for a null mutation in *Prkar1a* present unregulated PKA activity which results in severely reduced male fertility (Burton et al., 2006). Heterozygous mutant males show low sperm count, and mature sperm from the cauda epididymis present aberrant shapes, bended and fragmented tails, detached heads, and an inability to bind to the zona or fuse with zona-free oocytes upon *in vitro* insemination (Burton et al., 2006). It is shown that altered phosphorylation levels of nuclear proteins by PKA lead to stage-I round spermatids developing large nuclear areas devoid of chromatin, resulting in the aberrant phenotype (Burton et al., 2006). Of note, a similar fertility phenotype is observed in Carney complex patients, with sperm presenting abnormal heads and tails comparable to the mutant mice (Burton et al., 2006).

## Sperm Motility and Capacitation

Sperm motility and capacitation are key factors that permit the sperm to travel through the female genital tract, transverse the oocyte vestments (which include the cumulus mass and the zona pellucida, an extracellular glycoprotein matrix surrounding mammalian oocyte), and undergo acrosome exocytosis (Puga Molina et al., 2018; Ritagliati et al., 2018; Bhakta et al., 2019). During sperm capacitation, several modifications including alteration of motility and the induction of acrosomal exocytosis enable sperm to efficiently travel and penetrate the zona pellucida of the egg (Puga Molina et al., 2018). The motility of the sperm is ensured by the flagellar dyneins, the molecular motors that regulate shearing and bending of the sperm flagellum (Neesen et al., 2001). Also, the flagellum is defined by three functional regions, which are the mid-piece, the principal piece, and an end-piece. The principal piece presents the vast majority of characterized ion channels that regulate sperm motility (Lishko and Mannowetz, 2018).

Crucial molecular regulators of sperm motility and capacitation are Dynein Axonemal Heavy Chain 1 (*DNAH1*), the Cystic Fibrosis Transmembrane Conductance Regulator (*CFTR*), the CatSper channel, Glutathione peroxidase 4 (*GPx4*), Nephrocystin 4 (*NPHP4*, coding for Nephroretinin), PKD2, and the solute carrier family 39 member 4 *(SLC39A4*, which encodes a zinc transporter protein known as Zip4).

**FIGURE 2:**
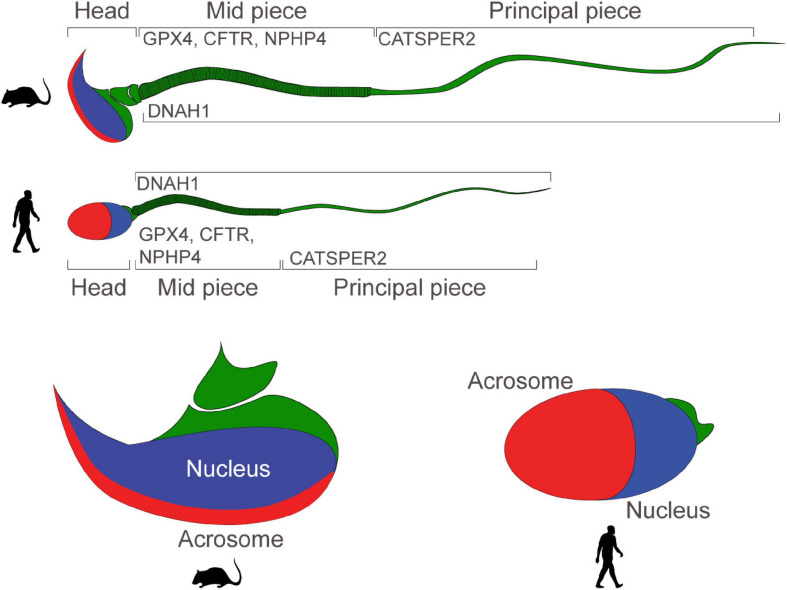
Motility and functionality of mouse and human sperm: schematic portraying the structure of mature mouse and human sperm. Proteins regulating sperm motility and ability to fertilize eggs are expressed in the mid-piece, or in the principal piece. Sperm heads present a nucleus and an acrosome.

DNAH1 is a central component of the dynein chains in the flagella of sperm, CFTR and CatSper are ion channels which mediate sperm capacitation prior to fertilization, GPX4 preserves normal mitochondrial function to ensure morphology and sperm progressive motility, Nephroretinin localizes next to the basal bodies of sperm flagella to regulate flagellar motility, PKD2 (described above) regulates directional sperm motility *in vivo* (in *Drosophila*), and ZIP4 regulates zinc uptake which has been reported to be critical during spermatogenesis and in the regulation of sperm motility (in the Japanese eel and in *C. elegans*) (Figure 2).

*DNAH1* encodes a heavy chain localized to the inner dynein arms of mammalian cilia and flagella. Cilia are comprised of outer and inner dynein arms, which contain light, intermediate, and heavy dynein chains (Neesen et al., 2001). Heavy dynein chains drive the motor function of cilia and flagella through ATP hydrolysis performed in the P loops of the heavy chain. On the N terminus of the heavy chains is located a stem structure which interacts with light and intermediate chains to create bonds and form dynein complexes (Neesen et al., 2001). On the C-terminus, a microtubule binding site serves as a point of attachment for motor function (Neesen et al., 2001). In mice, expression of the *DNAH1* homolog, *Mdhc7*, is observed in developing testis by postnatal day 17. Knockout mice have been generated through disrupting exons responsible for encoding the ATP binding site on dynein heavy chain 7 (Neesen et al., 2001). While heterozygotes do not possess notable impairments to fertility, homozygous knockout males are infertile and fail to produce MDHC7 protein. The majority of mutant sperm are immotile, indicating that the MDHC7 protein is an important component of dynein chains in the sperm flagella (Neesen et al., 2001). However, knockout mouse sperm can fertilize eggs *in vitro*, albeit with low efficiency, though no analyses of sperm-egg binding have been reported (Neesen et al., 2001). In humans, missense or frameshift mutations in *DNAH1* have been associated with ***Primary Ciliary Dyskinesia (PCD*)**. PCD manifests as respiratory deficits in affected patients causing sinusitis, bronchitis, and lung damage. Impaired ciliary function disrupts the ability of cilia in the airway to propel mucus (Imtiaz et al., 2015). Impairments to ciliary and flagellar function caused by PCD lead to subfertility in female patients due to reduced functionality of human oviduct cilia. Similarly to mice, infertility associated with PCD in men is correlated with defective sperm motility (Imtiaz et al., 2015); particularly, abnormalities in sperm flagella morphology are observed in patients with homozygous frameshift and nonsense mutations in *DNAH1*. Patients exhibit asthenzoospermia and flagellar morphological abnormalities such as short, coiled, kinked, and absent flagella (Wang et al., 2017). Sperm viability in affected patients ranges from 35 to 75% (Wang et al., 2017).

CFTR, a cAMP-modulated Cl^–^ channel, controls ion exchange and water secretion/absorption in epithelial tissues. Phosphorylation of the regulatory domain, ATP-binding to the nucleotide-binding domains, or ATP hydrolysis, controls channel activation, and loss-of-function mutations in *CFTR* result in cystic fibrosis (Riordan et al., 1989), the most common genetic disorder in Caucasians (1 individual affected over 3,500 newborns) (Castellani et al., 2009). Absence or defective CFTR leads to reduced epithelia permeability to Cl^–^, hence, reduced Cl^–^ secretion and a decrease of salt on the apical surface ensues, and these conditions fail to recall water to enter the lumen. Also, lack of CFTR leads to non-controlled activities of other channels, such as ENaC, which results in an unregulated absorption of salt from the airway surface liquid. The mucous blanket overlying the epithelia becomes thicker and compresses the cilia, thus delaying mucociliary clearance (Matsui et al., 1998), causing chronic lung infection and inflammation, pancreatic insufficiency, and infertility (Rowe et al., 2005). Indeed, ∼98% of male patients present infertility, mainly due to a congenital bilateral absence of *vas deferens*, leading to obstructive azoospermia (Chillón et al., 1995), although a number of cases have shown non-obstructive azoospermia, oligospermia, asthenospermia, and teratospermia (Chen et al., 2012). Of note, CFTR is expressed in mouse and human sperm (Hernández-González et al., 2007); sperm capacitation is defined by a series of physiological changes that the fertilizing sperm must acquire to fuse with an egg. Mouse and human sperm undergo a capacitation-associated plasma membrane hyperpolarization, which is necessary for the induction of acrosome exocytosis. Of interest, when CFTR is blocked with diphenylamine-2-carboxylic acid, the capacitation-associated hyperpolarization of the sperm membrane is repressed; on the other hand, exposure of sperm to genistein (a CFTR channel activator) induces sperm hyperpolarization under conditions that would not normally support capacitation (Hernández-González et al., 2007). No male germline conditional knockout mouse has been generated thus far, so it has not been possible to test the role of CFTR in spermatogenesis or sperm capacitation prior to fertilization.

CatSper is a flagellar specific and Ca^2+^-selective channel, which is defined by at least nine different protein –coding genes (*CatSper1–4*, β, γ, δ, ε, and ζ). CatSper1-4 delineates a pore-forming α subunit (Ren et al., 2001; Quill et al., 2003; Qi et al., 2007; Strünker et al., 2011), whereas the transmembrane CatSperβ, CatSperγ, CatSperδ, CatSperε, and cytosolic CatSperζ preserve proper channel structure and function (Liu et al., 2007; Wang et al., 2009; Chung et al., 2011, 2017). CatSper controls sperm hyperactivation by regulating Ca^2+^ signaling through the establishment of nanodomains that are localized throughout the sperm (Chung et al., 2014). Humans homozygous for a deletion in Chromosome 15 spanning *STRC* and *CATSPER2* genes have been associated with ***Deafness-infertility syndrome (DIS)***, which presents itself as an early-onset deafness in males and females with modest symmetric sensorineural hearing loss (Dgany et al., 2002, p. 1); men present normal ejaculate volume, though sperm count, motility, and morphology are affected, which results in asthenoteratozoospermia and male infertility (Dgany et al., 2002, p. 1). Male mice null for 8 of the 9 different protein–coding genes present sperm unable to undergo hyperactivation and fertilize eggs *in vitro* or *in vivo*. In *CatSperζ* null males, the channel is still functional albeit that CatSper nanodomains are disrupted, resulting in subfertility (Chung et al., 2011, 2014, 2017). CatSper Ca^2+^ nanodomains regulate phosphorylation cascades in the sperm tail, which modify the axonemal motion while initiating the peculiar asymmetric/high-angle bend of hyperactivated tail motility (Chung et al., 2014). Protein tyrosine phosphorylation increases in a time-dependent fashion during sperm capacitation (Visconti et al., 1995), with the majority of tyrosine phosphorylation observed in the axoneme and absence of CatSper leading to an increase of tyrosine phosphorylation, which also mislocalizes to periaxonemal regions (Chung et al., 2014). The inability of sperm to undergo hyperactivation, together with the disruption of the capacitation-initiated tyrosine phosphorylation pathway, makes sperm unable to fertilize eggs *in vivo* or *in vitro* (Chung et al., 2014).

*GPX4*, a member of the selenoproteins glutathione peroxidase family, uses glutathione as an electron donor to catalyze the reduction of hydroperoxides in membrane lipids (e.g., phospholipids, cholesterol, and cholestryl ester) (Brigelius-Flohé, 1999). GPX4 preserves normal mitochondrial function and suppresses apoptosis, while repairing oxidation damage to cardiolipin (a phospholipid constituting ∼20% of mitochondria lipid composition) in a number of organs, including male gonads. Gene deletion of *GPX4* in null mice leads to post-implantation embryo lethality (E7.5) (Imai et al., 2003). Tamoxifen-inducible inactivation of *GPX4* in neuron-specific conditional knockout newborn mouse pups leads to 12/15-lipoxygenase-derived lipid peroxidation, which in turn triggers apoptosis-inducing factor (AIF)-mediated cell death. Neuron-specific *GPX4* depletion leads to severe neurodegeneration with presence of pyknotic cells in the pyramidal layer of the CA3 region of the hippocampus and pups were euthanized on postnatal day 13 (Seiler et al., 2008). In humans, loss-of-function mutations (i.e., frameshift or nonsense) in the *GPX4* gene are associated with the manifestation of ***Spondylometaphyseal dysplasia Sedaghatian type (SMDS)***. SMDS is a rare disease that leads to neonatal death due to respiratory failure. This genetic condition is defined by severe metaphyseal chondrodysplasia, delayed epiphyseal ossification, platyspondyly, irregular iliac crests, and pulmonary hemorrhage. Moreover, defective development of the central nervous system is observed, and in particular abnormal neuronal migration, agenesis of the corpus callosum, pronounced frontotemporal pachygyria, simplified gyral pattern, partial lissencephaly, and severe cerebellar hypoplasia (Smith et al., 2014). GPX4 expression is also enriched in the mitochondria of testis and sperm. A spermatocyte-specific *GPX4* knockout mouse line shows male infertility due to oligoasthenozoospermia, and decreased sperm progressive motility associated with impaired mitochondrial membrane potential and cellular abnormalities (e.g., hairpin-like flagella bend at the midpiece, swelling of mitochondria) (Imai et al., 2009). *GPX4* appears to play a role in human spermatogenesis as well: a significant reduction in *GPX4* expression is found in a number of infertile oligospermic men (Imai et al., 2001).

*NPHP4* encodes a 1426 amino acid-long protein (also known as Nephroretinin), presenting a proline-rich domain between amino acids 458–514. NPHP4 localizes to the basal bodies of the primary cilia in polarized epithelial tubular cells, in the proximity of the cortical actin cytoskeleton, and to the centrosomes of dividing cells (Mollet et al., 2002). Molecular binding partners of NPHP4 are NPHP1, RP GTPase regulator interacting protein 1 (RPGRIP1) (Roepman et al., 2005), NPHP8 (alias of RPGRIP1L) (Delous et al., 2007) and Retinitis pigmentosa GTPase regulator (RPGR) (Murga-Zamalloa et al., 2010), proteins that regulate ciliary function. Homozygous missense or heterozygous frameshift mutations in *NPHP4* have been associated with ***Nephronophthisis***, an autosomal recessive kidney disease mainly affecting children and young adults (Strauss and Sommers, 1967) that presents with polyuria, polydipsia, and anemia (Wolf, 2015); it has been associated with different syndromes, including the Senior–Loken syndrome (SLSD) with retinitis pigmentosa (RP), Joubert syndrome with RP, cerebellar and brainstem malformations, and intellectual disability (Otto et al., 2002, 2005; Parisi et al., 2004). Mutant mice homozygous for a nonsense mutation in the *Nphp4* gene present male infertility (Won et al., 2011) with reduced sperm counts (98% reduction compared to fertile controls), scarce motility, and virtually absent progressive motility (Won et al., 2011). Electron microscopy of cross-section and longitudinal images of the flagella reveal intact normal nine microtubule doublets encircling a central microtubule pair in the mutant flagellar axoneme. NPHP4 normally localizes next to the basal bodies in the testicular spermatozoa and spermatids, and its absence seems to be the cause of the defective phenotype (Won et al., 2011): these sperm are unable to fertilize eggs *in vitro* or *in vivo* (Won et al., 2011).

Of note, PKD2 (associated with ADPKD, described above) regulates directional sperm motility in *Drosophila*, and null males present normal spermatogenesis but are unable to localize the egg *in vivo* (Gao et al., 2003).

*SLC39A4* encodes a zinc transporter protein known as Zip4 which is expressed in the human intestine, colon, ovary, and testis (Küry et al., 2002; Wang et al., 2004; Dufner-Beattie et al., 2007). In humans, missense mutations in *SLC39A4* affecting the ZIP4 transmembrane domains have been associated with ***Acrodermatitis enteropathica*** (***AE***), a genetic condition characterized by concurrent manifestation of diarrhea, dermatitis, alopecia, skin lesions, growth retardation, impaired wound healing, and male hypogonadism together with low sperm count (Maverakis et al., 2007) due to defective cellular zinc uptake. Deletion of *Slc39a4* in mice results in embryonic lethality (by embryonic day 10) (Dufner-Beattie et al., 2007), whereas heterozygous null mice present incomplete lethality prior to weaning. Those that survive show hydrocephalus, incomplete eye development, and growth retardation (Dufner-Beattie et al., 2007). In humans, hormone replacement therapy restores sperm count, improves seminiferous tubule volume, and stimulates spermatogenesis in azoospermic patients (Vicari et al., 1992). The role of zinc in mammalian spermatogenesis and fertilization has not been well described, however, it has been shown that zinc is necessary for proper spermatogenesis in the Japanese eel (*Anguilla japonica*), during which it accumulates in the mitochondria of spermatogonia and developing sperm and regulates sperm motility (Yamaguchi et al., 2009). Also, zinc controls sperm activation in *C. elegans* by inducing immotile spermatids to rapidly develop into mature, motile sperm (Zhao et al., 2018).

## Sperm Passage Through the Epididymis

The epididymis is defined by four anatomical regions, the initial segment: the caput, the corpus, and the cauda, the latter being the region connected to the *vas deferens*. Each of these regions present a tissue-enriched gene expression profile that is important to ensure proper concentrations of luminal ions, which are important for the maturation of the fertilizing sperm (Belleannée et al., 2012).

The Solute Carrier Family 22 Member 5 (*SLC22A5*) gene codes for the Organic Cation Transporter 2 (OCTN2), a transporter of organic cations and carnitine (Wu et al., 1999). OCTN2 normally transports an array of organic cations, however, its affinity to carnitine and the ability to transport carnitine increases in the presence of sodium (Wu et al., 1999). OCTN2 is expressed in the kidneys and other tissues including the heart, placenta, brain, testis, and epididymis (Wu et al., 1999; Rodríguez et al., 2002). In humans, homozygous deletions, splice-site, or frameshift mutations in *SLC22A5* have been associated with ***Primary systemic carnitine deficiency* (*SCD*)**, an autosomal recessive disorder characterized by negligible cellular uptake of carnitine in different tissues leading to progressive cardiomyopathy, skeletal myopathy, hypoglycaemia, and hyperammonaemia (Nezu et al., 1999). To become functionally mature and capable of fertilizing eggs, mammalian sperm transverse the epididymis, where glycoproteins, lipids and inorganic compounds assist with the maturation of sperm (Gervasi and Visconti, 2017). During sperm transport through the epididymis, carnitine acts as cofactor for the transport of long-chain fatty acid in the mitochondria; here, long-chain fatty acid undergoes beta-oxidation, producing acetyl-CoA, which is important for sperm to acquire motility in the distal epididymis (Dacheux and Dacheux, 2014). A mutant mouse (the juvenile visceral steatosis or *jvs* mouse) homozygous for a missense mutation in *Slc22a5* that abrogates carnitine transport (Nezu et al., 1999), presents an anatomically deformed epididymis and male infertility (Toshimori et al., 1999); in particular, the duct of the proximal epididymis accumulates an anomalous number of immature sperm, which occasionally causes the rupture of the epididymal epithelium, with sperm being reversed into the stroma (Toshimori et al., 1999). These phenotypic consequences lead to obstructive azoospermia, which is reversible with exogenous administration of L-carnitine (Toshimori et al., 1999).

*PKD1* (associated with ADPKD, described above) expression is also enriched in the epididymis (Nie and Arend, 2013). In mice, *Pkd1* null male mice present cystic dilation of the efferent ducts (derivatives of the mesonephric tubules), with the development of the epididymis being severely delayed or arrested (Nie and Arend, 2013).

## Conclusion

By the age of 25, approximately 5.3% of live-born humans develop genetic diseases (Baird et al., 1988), some of which result from deleterious mutations in genes that also regulate different aspects of gametogenesis and fertilization; plausibly the role of these genes in reproduction may be overlooked, as genetic conditions oftentimes are so severe that associated fertility disorders can go disregarded. Moreover, the clinical manifestation of a number of disorders have been reported after the appearance of a fertility phenotype (Eisenberg et al., 2014, 2016; Tarín et al., 2015). Thus, certain defective fertility phenotypes may represent a marker for future severe conditions. For example, among the conditions reviewed here, ADPKD incidence peak has been reported between the ages of 30 and 40 (Pei and Watnick, 2010); hence, an early diagnosis of male infertility associated with azoospermia or necrospermia (Nie and Arend, 2013, p. 1), or Sertoli cell only syndrome (Nygaard et al., 2015) could represent a possible red flag for a later onset of the ADPKD (Pei and Watnick, 2010) and genetic testing might be required. To understand better the genetic intersection between rare disorders and defective spermatogenesis, one could establish mouse lines expressing the exact mutation that has been associated with the aberrant phenotypes in humans. Indeed, as an increasing number of individual human genomes has lately become available (1000 Genomes Project Consortium et al., 2015; Wang et al., 2020), the functional characterization of novel genes (Kiyozumi et al., 2020; Noda et al., 2020), or putative deleterious variants (Singh and Schimenti, 2015) in transgenic animal models, will allow us to identify more comorbidities between gametogenesis or fertilization defects and rare genetic disorders (Vaquer et al., 2013; Hmeljak and Justice, 2019).

## Author Contributions

EL and MA conceived the study and wrote the manuscript. EL, LG, HB, and CG performed the database analyses and literature research. All the authors contributed to the article and approved the submitted version.

## Conflict of Interest

The authors declare that the research was conducted in the absence of any commercial or financial relationships that could be construed as a potential conflict of interest.
